# Contrasting Patterns of Genetic Differentiation among Blackcaps (*Sylvia atricapilla*) with Divergent Migratory Orientations in Europe

**DOI:** 10.1371/journal.pone.0081365

**Published:** 2013-11-21

**Authors:** Raeann Mettler, H. Martin Schaefer, Nikita Chernetsov, Wolfgang Fiedler, Keith A. Hobson, Mihaela Ilieva, Elisabeth Imhof, Arild Johnsen, Swen C. Renner, Gregor Rolshausen, David Serrano, Tomasz Wesołowski, Gernot Segelbacher

**Affiliations:** 1 Department of Evolutionary Biology and Animal Ecology, University of Freiburg, Freiburg, Germany; 2 Biological Station Rybachy, Zoological Institute, Rybachy, Kaliningrad Region, Russia; 3 Max Planck Institute for Ornithology, Vogelwarte, Radolfzell, Germany; 4 University of Konstanz, Department of Biology, Konstanz, Germany; 5 Environment Canada, Science and Technology Branch, Saskatoon, Saskatchewan, Canada; 6 Institute of Biodiversity and Ecosystem Research, Bulgarian Academy of Sciences, Sofia, Bulgaria; 7 Wildlife Ecology and Management, University of Freiburg, Freiburg, Germany; 8 Natural History Museum, University of Oslo, Oslo, Norway; 9 Institute of Experimental Ecology, Ulm University, Ulm, Germany; 10 Smithsonian Conservation Biology Institute, Front Royal, United States of America; 11 Redpath Museum & Department of Biology, McGill University, Montreal, Quebec, Canada; 12 Department of Conservation Biology, Estación Biológica de Doñana (EBD-CSIC), Sevilla, Spain; 13 Laboratory of Forest Biology, Wrocław University, Wrocław, Poland; University of Milan, Italy

## Abstract

Migratory divides are thought to facilitate behavioral, ecological, and genetic divergence among populations with different migratory routes. However, it is currently contentious how much genetic divergence is needed to maintain distinct migratory behavior across migratory divides. Here we investigate patterns of neutral genetic differentiation among Blackcap (*Sylvia atricapilla*) populations with different migratory strategies across Europe. We compare the level of genetic divergence of populations migrating to southwestern (SW) or southeastern (SE) wintering areas with birds wintering in the British Isles following a recently established northwesterly (NW) migration route. The migratory divide between SW and SE wintering areas can be interpreted as a result of a re-colonization process after the last glaciation. Thus we predicted greater levels of genetic differentiation among the SW/SE populations. However, a lack of genetic differentiation was found between SW and SE populations, suggesting that interbreeding likely occurs among Blackcaps with different migratory orientations across a large area; therefore the SW/SE migratory divide can be seen as diffuse, broad band and is, at best, a weak isolating barrier. Conversely, weak, albeit significant genetic differentiation was evident between NW and SW migrants breeding sympatrically in southern Germany, suggesting a stronger isolating mechanism may be acting in this population. Populations located within/near the SW/SE contact zone were the least genetically divergent from NW migrants, confirming NW migrants likely originated from within the contact zone. Significant isolation-by-distance was found among eastern Blackcap populations (i.e. SE migrants), but not among western populations (i.e. NW and SW migrants), revealing different patterns of genetic divergence among Blackcap populations in Europe. We discuss possible explanations for the genetic structure of European Blackcaps and how gene flow influences the persistence of divergent migratory behaviors.

## Introduction

Patterns of geographic isolation during the Pleistocene in temperate areas have allowed behavioral, ecological, and genetic divergence among geographically isolated populations in a range of taxa [1,2]. However, panmixia is often found within highly mobile vertebrate populations, despite presumed geographic barriers to dispersal and divergent migratory pathways [3–5]. While geographic barriers are instrumental in the formation of initial reproductive isolation, further isolating barriers are needed for continued genetic divergence in the face of gene flow, for example in the case of secondary contact between previously isolated populations at contact zones [6].

Migratory divides represent the locations at which populations maintain divergent migratory routes, often originating during periods of allopatry, but can also evolve into hybrid zones as a result of secondary contact and interbreeding between populations [7]. If isolating barriers are sufficiently strong to maintain some degree of reproductive isolation between divergent populations, the level of genetic divergence among populations with distinct migratory routes may be high [8]. However, migratory divides are also known to be leaky barriers to gene flow, despite divergent migratory routes utilized by organisms, such as those found in migratory butterflies [9] and birds [10,11]. Where interbreeding does occur between different migrant types, it is hypothesized that migratory divides can also represent hybrid zones maintained by selection against hybrids as a form of reproductive isolation [12–14]. While migratory behavior does not appear to be a strong isolation barrier, it is among the best examples of a mechanism promoting adaptive microevolution [15,16]. For example, assortative mating based upon allochronic spring arrival to the breeding grounds can facilitate adaptive microevolution in passerines and promote genetic divergence within sympatric breeding populations [17–20] . 

The Blackcap (*Sylvia atricapilla*) is a common European passerine and serves as a model species to study the evolution of migratory behavior because it spans the full spectrum of migratory distances (from sedentary to long-distance migrations) and migratory orientations. Several migratory traits have been shown to be under genetic control in Blackcaps, including timing of migration, direction, and duration/length of migration [21–24], while moderate-to-high heritabilities have been found for some of these traits in both wild and experimental populations [15,22]. In central Europe, Blackcaps exhibit a southwest/southeast (SW/SE) migratory divide resulting from the geographic isolation of potential glacial refugia in the Iberian Peninsula and Balkan Peninsula during the Pleistocene, followed by post-glacial expansion [21,25–27]. Weak but significant genetic structure in mitochondrial DNA (mtDNA) has been found to occur between Blackcap populations sampled on opposing sides of this divide [28].

**Figure 1:**
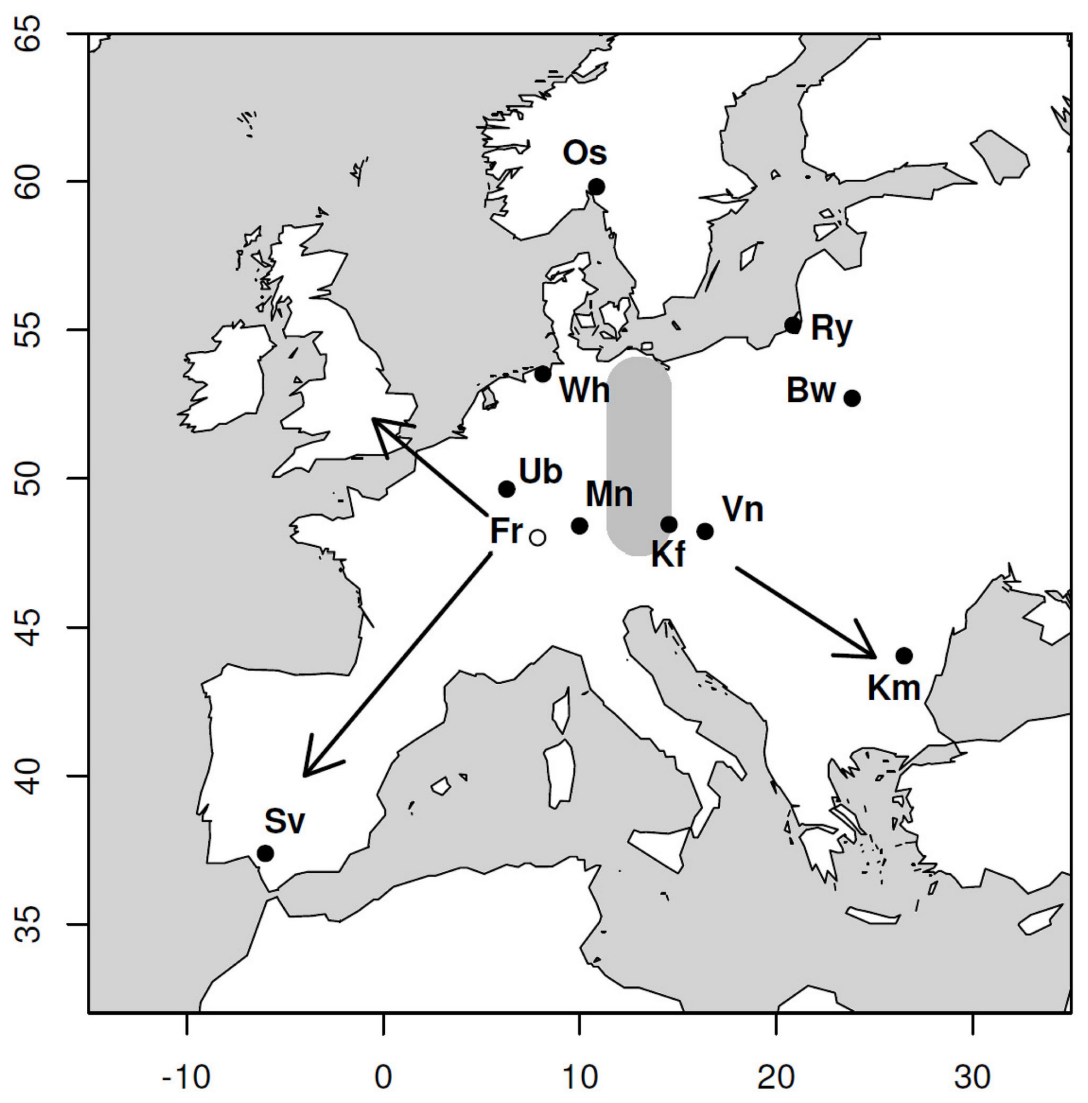
Sampling localities of Blackcaps captured in Europe. Population codes: **Sv** = Sevilla, ES; **Ub** = Uebersyren, LU; **Fr** = Freiburg, DE; **Wh** = Wilhelmshaven, DE; **Mn** = Münsingen, DE; **Os** = Oslo, NO; **Kf** = Kefermarkt, AT; **Vn** = Vienna, AT; **Ry** = Rybachy, RU; **Bw** = Białowieża Forest, PL; **Km** = Kalimok, BG. Individuals collected in Freiburg, Germany (Fr), indicated with the open symbol, were divided into populations of NW and SW migrants by stable isotope *δ* ^2^H values in claws. The thick, grey band represents approximate location of SW/SE migratory divide, centered at 13°E (according to[25,27,50]). Degrees East and North are shown on the X and Y axes, respectively.

As recently as during the latter half of the last century, a portion of SW migrant Blackcaps shifted their autumn migration route to the northwest (NW), overwintering on the British Isles. It has been hypothesized by Helbig [27] that these NW migrants originated from within the SW/SE contact zone. Currently a novel northwest/southwest (NW/SW) migratory polymorphism exists where these different migrants breed sympatrically in southern Germany [29]. This change in orientation has facilitated rapid morphological, behavioral, and genetic divergence between migrants from different wintering grounds and may promote assortative mating as an isolating barrier in sympatry [17,18,20,30]. In this study, we investigate genetic variation in 12 populations of wild Blackcaps using 14 polymorphic microsatellite loci. Sampling sites spanned both the historical SW/SE migratory divide in central Europe and the novel NW/SW migratory polymorphism present in southern Germany (Figure 1). By simultaneously characterizing levels of neutral genetic differentiation across the SW/SE migratory divide as well as the NW/SW polymorphism, we investigated two competing hypotheses: 1) if the migratory divide represents an adequate isolating barrier, genetic divergence between SW and SE migrants should be detectable (as found in [28]) and, given the long history of this divide, would be larger than that found at the novel NW/SW migratory polymorphism; 2) if the migratory divide is an incomplete barrier to gene flow, levels of genetic divergence should be lower across the SW/SE migratory divide (as found in other European passerines, [12,31]) compared to the novel NW/SW migratory polymorphism experiencing rapid, adaptive divergence in sympatry [17,18,20]. 

## Methods

### Ethics Statement

Samples from the Schwäbische Alb (Münsingen) and Freiburg have been collected under field work and animal experiment permits granted by the responsible state environmental offices of Baden-Württemberg (RPT Tierversuch-Nr. 1056 and 55-8853.17/0, respectively). Samples collected in Kefermarkt, Austria were approved from the district Freistadt permit Nr. N10-75-2010. Sampling in Rybachy (Russia) was performed under the license from Kaliningrad Regional Agency for Protection, Reproduction and Use of Animal World and Forests. Trapping and sampling permits in Spain were granted by the Consejería de Medio Ambiente of the government of Andalucía and in Bulgaria under permit N243/1.03.2010 from Bulgarian Ministry of Environment and Waters. 

### Sample collection and allocation

Blood samples from 502 Blackcaps were collected at 12 sample sites across Europe from 2001 - 2011 (Figure 1 and Table 1). Individuals captured in Sevilla, Spain (Sv) during the non-breeding season (January) were determined to be overwintering, short-distance migrants based on two lines of evidence: 1) Sevilla birds had relatively short wings compared to other breeding populations in central Europe, thus travelled a shorter distance during migration, and 2) they were found to be genetically differentiated (at microsatellite loci) from resident Spanish Blackcaps identified in a previous study [20]. The remaining 11 sites were sampled during the breeding season (Table 1). While we assume most to be breeding individuals (supported by our ringing data (data not shown)), we cannot rule out that some spring migrants may have been caught as well. All birds captured at a single sampling site were considered as populations in the following analyses. 

**Table 1:** Sampling locations of Blackcaps and summary statistics of population genetics.

**Code**	**Population**	***N***	**Lat**	**Long**	**Sampling Period**	**Year**	***A*_E_ (SE)**	***H*_O_(SE)**	***H*_E_ (SE)**	**MigDir**	**MigDist**
Sv	Sevilla, ES1	8	37.65	-5.58	25 Jan	2011	3.79 (0.40)	0.65 (0.06)	0.76 (0.02)	SW	short
Ub	Uebersyren, LU	43	49.63	6.28	25 Mar – 2 Apr	2011	4.33 (0.50)	0.72 (0.03)	0.74 (0.03)	SW	intermed
Fr_NW_	Freiburg, DE	23	48.03	7.82	23 Mar – 19 Apr	2011	4.15 (0.47)	0.66 (0.05)	0.73 (0.03)	NW	intermed
Fr_SW_	Freiburg, DE	82	48.03	7.82	23 Mar – 19 Apr	2011	4.62 (0.54)	0.70 (0.02)	0.76 (0.02)	SW	intermed
Wh	Wilhelmshaven, DE	24	53.52	8.12	Summer2	2007	4.17 (0.40)	0.60 (0.03)	0.75 (0.02)	SW	long
Mn	Münsingen, DE	25	48.40	10.00	16 Jun – 13 Jul	2011	4.32 (0.56)	0.72 (0.03)	0.74 (0.03)	SW	intermed
Os	Oslo, NO	25	59.84	10.86	4 May – 13 Jul	2001, 2004, 2008, 2010	4.11 (0.46)	0.68 (0.03)	0.73 (0.03)	SW	long
Kf	Kefermarkt, AT	105	48.44	14.54	14 Apr – 2 May	2011	4.63 (0.50)	0.69 (0.03)	0.76 (0.02)	SE	intermed
Vn	Vienna, AT	35	48.22	16.28	12 May – 21 May	2010	4.39 (0.50)	0.72 (0.02)	0.75 (0.03)	SE	intermed
Ry	Rybachy, RU	51	55.15	20.85	20 Apr – 24 Apr	2011	4.32 (0.52)	0.66 (0.03)	0.74 (0.03)	SE	long
Bw	Białowieża, PL	61	52.7	23.87	14 Apr – 6 May	2011	4.48 (0.45)	.73 (0.03)	0.76 (0.02)	SE	long
Km	Kalimok, BG	20	44.00	26.26	16 Apr – 7 Jun	2010, 2011	3.87 (0.38)	0.71 (0.04)	0.73 (0.03)	SE	long

***N***: sample size; **Lat**: decimal degree latitude; **Long**: decimal degree longitude; **Sampling Period**: dates samples collected; **Year**: year(s) samples collected; ***A*_e_**: effective number of alleles = 1 / (Sum pi^2), where Sum pi2 is the sum of the squared population allele frequencies; ***H*_o_**: observed heterozygosity; ***H*_e_**: unbiased expected heterozygosity = (2N / (2N-1)) x expected heterozygosity; (**SE**): standard error; **MigDir**: autumn migratory direction; **MigDist**: migratory distance ^1^. population sampled during the non-breeding season ^2^. precise sampling period information not available.

Stable isotope values from avian tissues can be used to discriminate between possible wintering grounds of migratory birds in Europe [32]. In particular, stable hydrogen isotope values (*δ* ^2^H) in keratins (claws and feathers) vary by latitude in Europe and have been previously used to distinguish which individuals winter at either northern or southern latitudes among sympatrically breeding Blackcaps in southern Germany [17,18,20]. To distinguish among NW and SW migrants breeding sympatrically in the Freiburg, Germany (Fr) population in this study, approximately 2 mm of claw material was collected from Blackcaps in the spring of 2011 and analyzed for *δ* ^2^H at the Environment Canada stable isotope facility in Saskatoon, Canada, using the comparative equilibrium method [33]. Claw *δ* ^2^H from eight Blackcaps caught in Sevilla, Spain (in Jan. 2011) for this study was used as a southern reference to identify SW migrants (mean *δ* ^2^H: -51.6‰, SD: 5.8). Due to the observed interannual variation in claw *δ* ^2^H and the absence of Blackcap reference claws from the British Isles during the 2010-2011 winter, *δ* ^2^H was analyzed from overwintering Blackcaps and other species (*Cyanistes caeruleus, Parus major, Fringilla coelebs, Prunella modularis, Carduelis spinus*) captured in Britain, Ireland and Scotland between 2002-2010 as part of this study (unpublished) and a previous study of Bearhop et al. [17]. We used the Bearhop et al. [17] estimate of overwintering *C. caeruleus* and *P. major* (2002-2003) as a northern reference (mean *δ* ^2^H: -94.3, SD: 4.9) and best approximation of the wintering ground signature of NW migrants in this study. Blackcaps caught in Freiburg in the spring of 2011 were assigned to wintering grounds using a 75% probability criterion based on reference claw *δ* ^2^H ( Figure S1), as previously described in Rolshausen et al. [20]. 

In addition to Freiburg, Blackcaps from three other collecting sites were analyzed for variation in claw *δ* ^2^H (Ub, Kf, and Bw – see Figure S1). The distributions of *δ* ^2^H were found to differ significantly among these four populations (Kruskal-Wallis *P* < 0.05). Apart from the Freiburg population (see above), only one individual caught in Uebersyren, LU was marginally categorized (78.4% probability) with a NW claw *δ* ^2^H value among the other populations tested; therefore, we found little indication of NW migrants present at these three additional locations during spring sampling. Due to the lack of longitudinal spatial resolution in claw *δ* ^2^H for birds in Europe, Blackcap populations (excluding Fr) were therefore categorized as SW or SE migrants using the approximate location of the SW/SE migratory divide in central Europe found in other studies [26–28] and published ring recoveries for this species [25,34–36] as classification criteria. The autumn migratory direction of Blackcaps breeding on the southern coast of Norway (Os) likely encompasses a broad front spanning SW – SE orientations [36,37] and so, while we initially analyzed this population as SW migrants, we also repeated the analyses by considering this population as SE migrants.

### PCR and genotyping

Genomic DNA was extracted from blood samples using DNeasy Blood and Tissue kits (Qiagen) following manufacturer’s protocol. A total of 14 microsatellite markers were utilized (Table S1), comprising nine markers developed for this study (Syla1, Syla2, Syla3, Syla12, Syla14, Syla15, Syla18, Syla19, Syla20 [38]) and five markers previously developed for this species (Syl1, Syl2, Syl6, Syl9, and Syl10 [39]). Microsatellite loci were amplified with polymerase chain reaction (PCR) for all individuals in 20µl reactions including: 1x Qiagen CoralLoad Concentrate, 1x Qiagen PCR Buffer (containing KCl and (NH_4_)_2_SO_4_), 0.25 µM labeled forward primer, 0.25 µM reverse primer, 200 µM of each dNTP, 1.25 U Qiagen Top Taq DNA polymerase, and 1.0µl DNA template. Mastercycler Gradient Thermocyclers (Eppendorf GmbH, Hamburg Germany) were used to run PCR under the following conditions: initial denaturing at 94 °C for 5 min, followed by 20 cycles of 94°C for 30 s, 60°C for 30 s (decreased by 0.5°C per cycle), and 72°C for 45 s, followed by 15 cycles of 94°C for 30 s, 50°C for 30 s, and 72°C for 45 s with a final extension of 72°C for 3 min. Forward primers were labeled with FAM or HEX florescent dyes (Applied Biosystems), PCR products were genotyped on an ABI3130xl Genetic Analyzer (Applied Biosystems), and genotypes were analyzed with PeakScanner v1.0 software (Applied Biosystems). Raw genotype sizes were rounded and binned according to repeat size using TANDEM [40]. The mean number of effective alleles (*A*_E_) and observed and expected heterozygosities (*H*_O_ and *H*_E_) were estimated for each population with GenAlEx v6.41[41]. Deviations from Hardy Weinberg Equilibrium (HWE), genotypic linkage disequilibrium (LD) between loci, and the frequency of null alleles were estimated with Genepop v4.1.2 [42]. Estimated *P*-values from HWE and LD tests were corrected for false discovery rate using the Qvalue program [43].

### Genetic structure

Pairwise genetic differentiation among Blackcap populations was estimated with the fixation index *D*_est_ (the unbiased estimator of *D*, [44]) using the R package “DEMEtics” [44,45]; complementary *F*_ST_ values estimated with Arlequin v3.5.1.3 [46] were found to be highly correlated with *D*_est_ values (Kendall’s tau = 0.637, *P* < 0.0001) and so, we report only *D*_est_ values hereafter. Locus Syl10 has been shown to deviate from HWE in a previous study [20]. We included this marker in our dataset as it has been proven to be highly variable. However, we ran *D*_est_ analyses including and excluding this marker for all comparisons and found that *D*_est_ values were highly correlated between analyses (Kendall’s tau = 0.842, *P* < 0.0001). Wilcoxon Sum Rank tests were implemented to investigate the independence of the distributions of *D*_est_ values within and among migratory orientations; the Uebersyren NW migrant identified via claw *δ* ^2^H was excluded from these analyzes.

Isolation-by-distance among Blackcap populations was explored with Mantel tests using Isolation by Distance Web Service v3.23 [47] to investigate the relationship between geographic distance and genetic distance: Euclidean geographic distance among all 12 populations was calculated with the R package “fossil” [48], pairwise genetic distance between populations was calculated as *D*_est_ /(1- D_est_) following Slatkin [49], and 10,000 randomizations were implemented. Additional Mantel tests were run for only those populations west of the SW/SE migratory divide and those populations east of the divide to investigate differential effects of isolation-by-distance on either side of the SW/SE divide.

The relationship between genetic distance and longitudinal geographic distance was investigated between Blackcap populations located on opposing sides of the SW/SE migratory divide, which was approximated at 13°E (Figure 1); we acknowledge that the contact zone between SW and SE migrants is potentially a broad, diffuse zone where migrants come into contact and here use this precise position as an approximate zone center (consistent with Blackcap ring recovery data [25–27,50]) for analytical purposes only. The latitude of each population was set to zero and the Euclidean (longitudinal) distance between populations was calculated with the R package “fossil” [48]. Spearman-Rank correlations were used to explore the relationship between genetic distance and longitudinal geographic distance between populations. To determine if the relationship between genetic distance and geographic distance was significantly different from random, measures of geographic distance were resampled 10,000x and tested against pairwise genetic distances with Spearman rank correlations. True *rho* values determined for genetic distance vs. geographic distance were then compared to confidence intervals constructed from random distributions to determine significance of true values. Statistical analyses were conducted with R statistical computing environment [51].

Genetic structure was further investigated from genotype data using a MCMC model-based clustering method implemented with STRUCTURE software v2.3.4 [52]. The most likely number of genetic clusters (*K*) was assessed assuming admixture and locprior of the individual populations and five iterations were run per *K* (Burn-in: 100,000; Repetitions: 500,000). 

### Hierarchical structuring of genetic variance

Populations were grouped by autumn migratory orientation (i.e. NW, SW, SE) and migratory distance (i.e. short-, intermediate-, long-distance) inferred from ring-recoveries (Table 1 and Figure 1; [25,35]). We investigated the effect of hierarchical structuring on the partitioning of genetic differentiation by categorizing individuals by migratory orientation, migratory distance, and the population level (in decreasing hierarchical order) using the R package “HierFstat” [53]. Here, the population level was nested within the level of migratory distance, and migratory distance was nested within the migratory direction level (population/distance/direction); the effect of each level was tested with 10,000 permutations to determine the level of significance of this structuring. Hierarchy A categorizes the Norwegian population (Os) with a SW migratory direction, while Hierarchy B categorizes the same population with a SE migratory direction. The Uebersyren NW migrant identified via claw *δ* ^2^H was excluded from these analyses.

## Results

### Genetic diversity

Observed multilocus population heterozygosities were high and ranged from 0.60 - 0.73, with a mean observed heterozygosity of 0.69 and mean number of effective alleles of 4.26 (Table 1). Only 18 of 168 total HWE tests indicated a deficiency of heterozygotes (*Q* < 0.01), although patterns were not consistent across populations (Table S1). The Syl10 locus deviated from HWE at a high frequency (i.e. in nine populations). In a global analysis including all populations, one comparison was in linkage disequilibrium (*Q* < 0.05: Syl2/Syl6), but found to be significant only in the Kefermarkt population when populations were analyzed separately. Overall we thus can assume independence between loci used in this study. Allele frequencies at each population per locus are listed in Table S2.

### Genetic structure

All birds could be assigned to one single population as STRUCTURE analysis identified the highest posterior probability for the number of genetic clusters to be *K*=1 (Table S3). Despite this lack of resolution in genetic structure, significant neutral genetic differentiation was evident among some population comparisons (Table 2). Most notably, genetic differentiation was significant between sympatric NW and SW migrants in Freiburg, with *D*_est_ = 0.029 (*F*_ST_ = 0.007). NW migrants breeding in Freiburg were also found to be significantly differentiated from allopatric populations in Uebersyren, Kalimok, and Sevilla, while SW migrants in Freiburg were undifferentiated from all allopatric populations. Freiburg NW migrants were most genetically similar to populations within/near the contact zone at the SW/SE migratory divide (Table 2).

**Table 2:** Pairwise genetic differentiation between sampled populations.

	**western populations**							**eastern populations**				
**Code**	**Sv**	**Ub**	**Fr_NW_**	**Fr_SW_**	**Wh**	**Mn**	**Os**	**Kf**	**Vn**	**Ry**	**Bw**	**Km**
**Sv**	-	0.057	0.059	0.035	0.017	0.029	0.020	0.022	0.014	0.051	0.038	0.008
**Ub**	**0.054**	-	0.028	0.004	0.004	0.000	0.000	0.005	0.000	0.001	0.000	0.041
**Fr_NW_**	**0.056**	**0.027**	-	0.030	0.030	0.008	0.020	0.007	0.009	0.025	0.020	0.071
**Fr_SW_**	0.034	0.004	**0.029**	-	0.018	0.000	0.000	0.000	0.004	0.010	0.001	0.021
**Wh**	0.017	0.004	0.029	0.017	-	0.009	0.000	0.004	0.000	0.013	0.000	0.032
**Mn**	0.029	0.000	0.008	0.000	0.009	-	0.000	0.004	0.000	0.005	0.005	0.025
**Os**	0.020	0.000	0.019	0.000	0.000	0.000	-	0.006	0.000	0.007	0.000	0.002
**Kf**	0.022	0.005	0.007	0.000	0.004	0.004	0.006	-	0.000	0.010	0.001	0.040
**Vn**	0.014	0.000	0.009	0.004	0.000	0.000	0.000	0.000	-	0.003	0.000	0.034
**Ry**	0.048	0.001	0.024	0.010	0.013	0.005	0.007	0.009	0.003	-	0.000	0.049
**Bw**	0.036	0.000	0.019	0.001	0.000	0.005	0.000	0.001	0.000	0.000	-	0.016
**Km**	0.008	**0.040**	**0.066**	0.020	0.031	0.025	0.002	**0.038**	**0.033**	**0.046**	0.016	-

Pairwise *D*_est_ values of genetic differentiation (below diagonal) and genetic distance (above diagonal) measured as *D*_est_ /(1- D_est_) following Slatkin (1995). Populations are sorted by longitude. **Western Populations**: Sv, Ub, Fr_NW_, Fr_SW_, Wh, Mn, Os. **Eastern Populations**: Kf, Vn, Ry, Bw, Km. Significance: *P* < 0.05 in bold; *P* < 0.01 in bold and underlined

**Figure 2:**
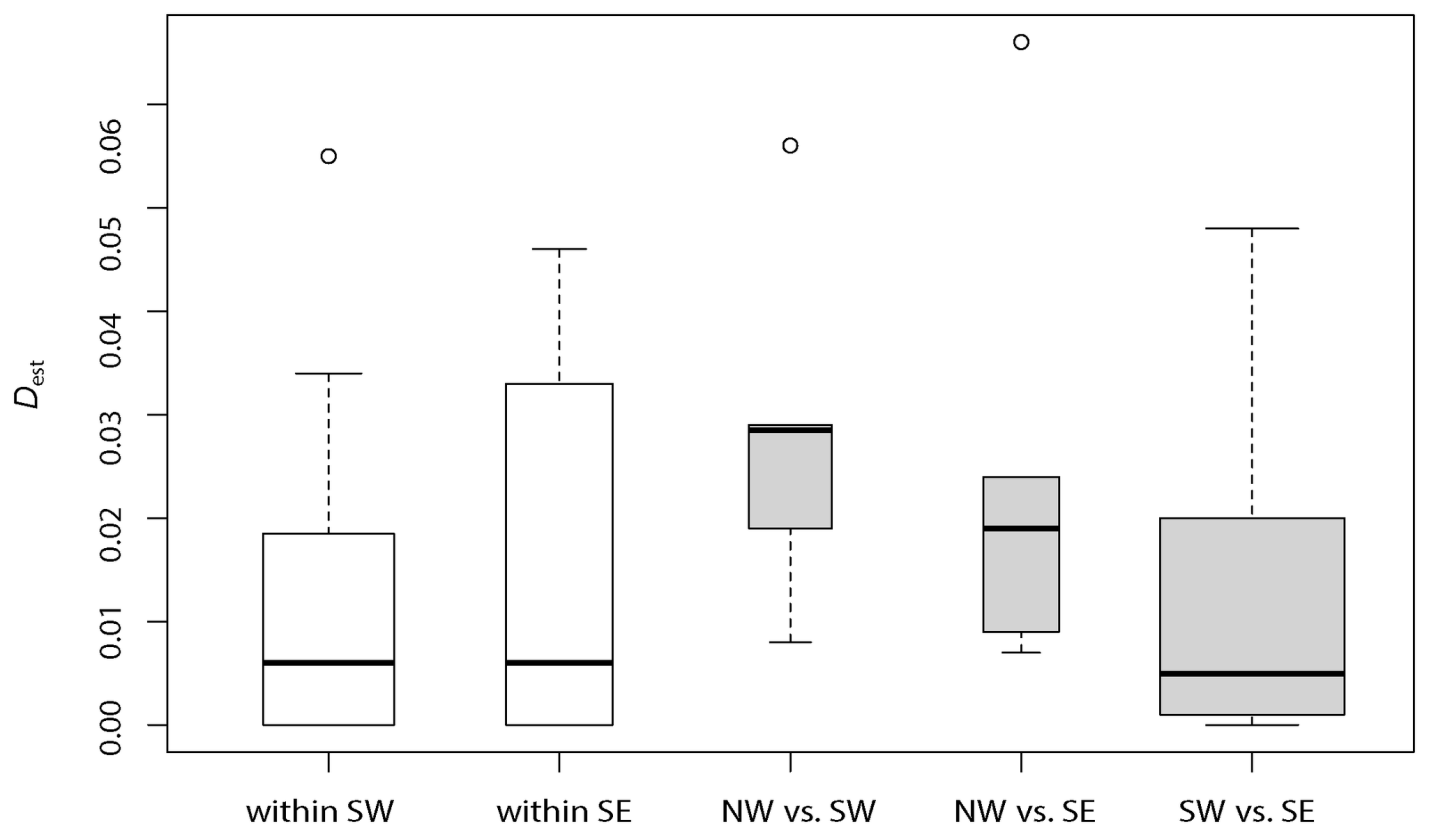
Distribution of *D*_*est*_ values among population comparisons. Comparisons within NW, SW, SE migratory orientations are shown as open bars and comparisons among NW, SW, SE migratory orientations are shown as shaded bars. Within SW orientations, *D_est_* comparisons did not differ from comparisons within SE orientations (Wilcoxon test *P* > 0.05). NW vs. SW comparisons were found to have significantly higher *D*_*est*_ values than SW vs. SE comparisons (Wilcoxon test *P* = 0.013). Remaining comparisons among groups were non-significant (*P* > 0.05). Width of bar is proportional to sample size.

Within the migrant groups, comparisons among SE migrants had higher *D*_est_ values than SW migrants, although distributions did not differ significantly between the groups (Wilcoxon test *P* > 0.05; Figure 2). Among the migrant groups, comparisons among NW vs. SW migrants had the highest *D*_est_ values and significantly differed from lower *D*_est_ values estimated from SW vs. SE migrant comparisons (Wilcoxon test *P* = 0.013; Figure 2). Levels of genetic differentiation among NW vs. SW comparisons did not significantly differ from NW vs. SE comparisons; similarly, NW vs. SE comparisons did not significantly differ from SW vs. SE comparisons (Wilcoxon tests *P* > 0.05; Figure 2). 

### Isolation-by-distance

**Figure 3:**
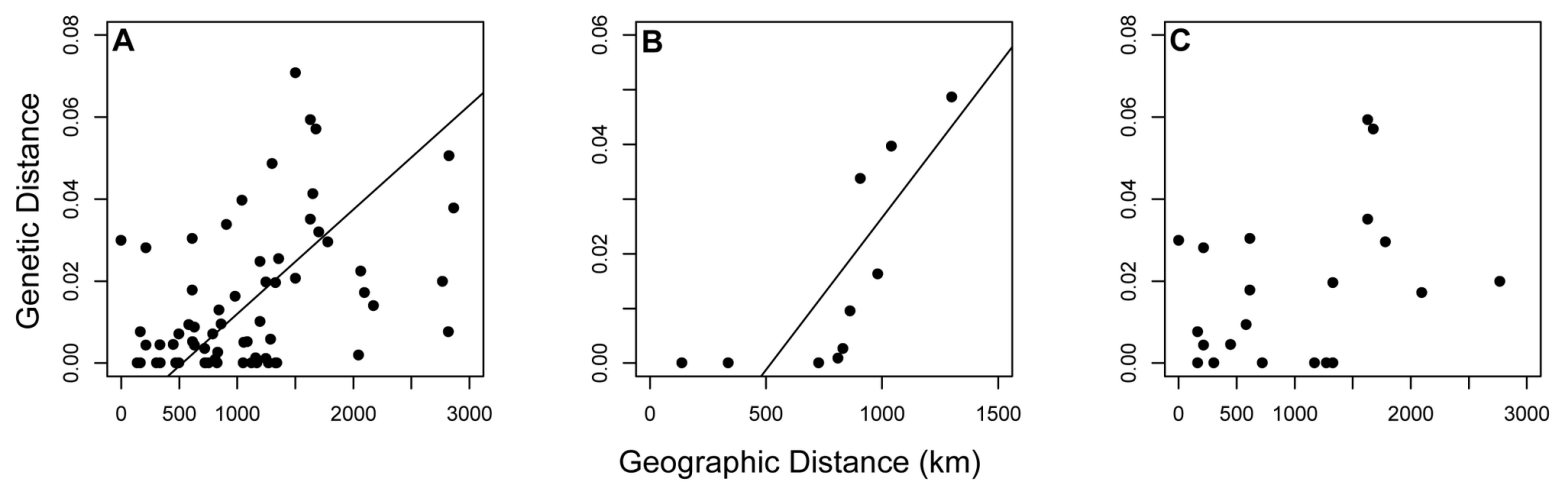
Isolation-by-distance results among Blackcap populations: the relationship between geographic distance (km) vs. *D_est_* genetic distance. (A) All populations included: *r* = 0.424, *P* = 0.045; (B) Eastern populations only: *r* = 0.744 *P* = 0.008; (C) Western populations only: *r* = 0.364, *P* = 0.174. Os population considered as SW migrants.

Mantel tests revealed significant isolation-by-distance among all 12 Blackcap populations (Figure 3A: *r* = 0.424, *P* = 0.045), although the relationship no longer occurred when the non-breeding, short-distance migrant population in Sevilla is excluded (*r* = 0.285, *P* = 0.117). Among the five eastern populations (i.e. those east of the SW/SE divide), isolation-by-distance was found significant (Figure 3B: *r* = 0.744, *P* = 0.008). Conversely, isolation-by-distance was non-significant among the seven western populations (Figure 3C: *r* = 0.364, *P* = 0.174), even when excluding the non-breeding Sevilla population (*r* = -0.275, *P* = 0.754). 

**Figure 4:**
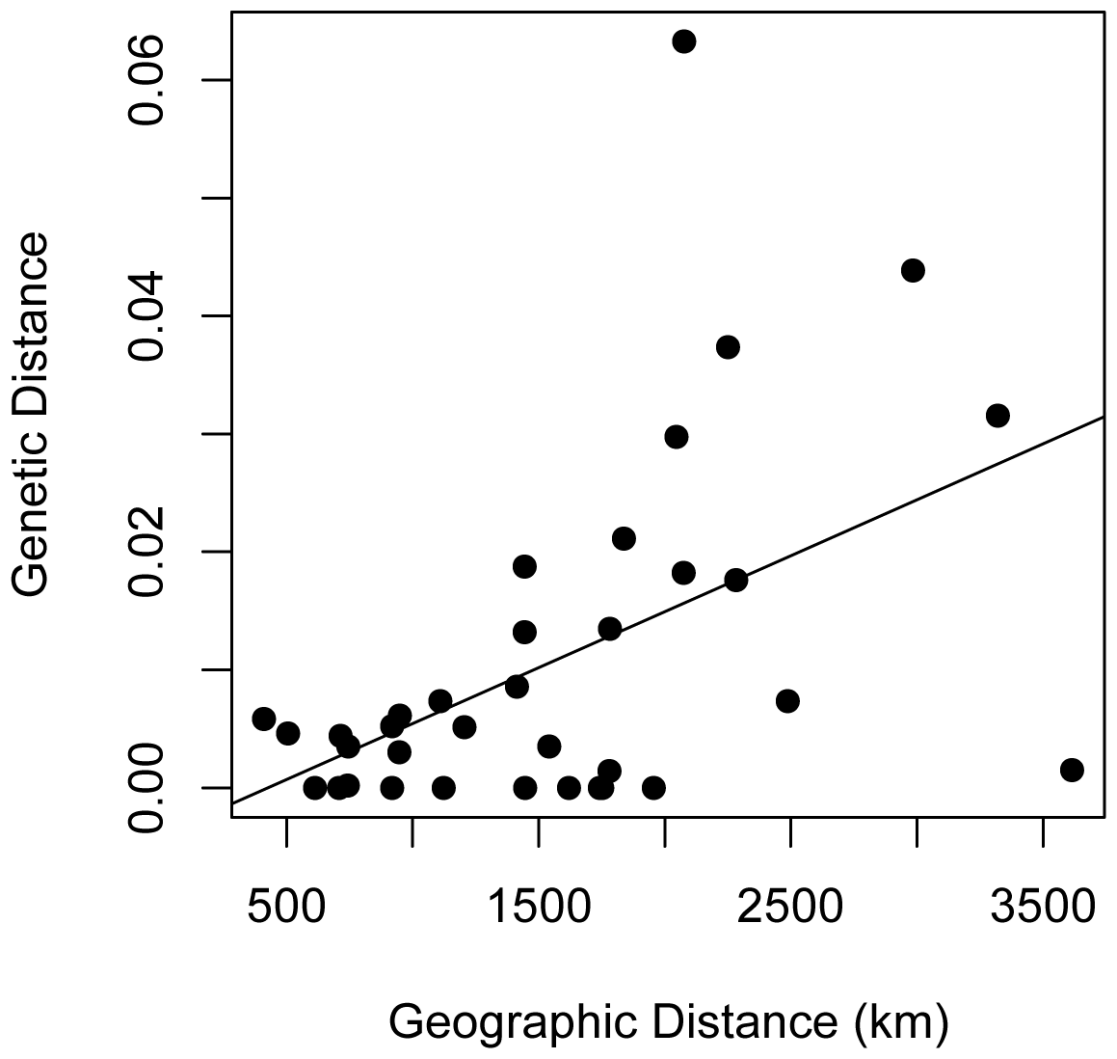
Relationship between geographic distance (km) and *D*_*est*_ genetic distance. Pairwise measurements estimated between population pairs located on opposing sides of the SW/SE migratory divide revealed a positive relationship (rho = 0.467, *P* = 0.005). All 12 populations included with the Os population considered as a ‘western’ population.

By exploring the relationship between genetic distance and the longitudinal geographic distance of population pairs located on opposing sides of the SW/SE divide, a strong, positive correlation was evident (Figure 4, rho = 0.467 *P* = 0.005). The least genetic distance was found between populations near the center of the SW/SE divide (approximated at 13°E) and genetic distance increased significantly with increased geographic distance between populations. For example, all pairwise *D*_est_ comparisons between Münsingen (Mn), Kefermarkt (Kf), and Vienna (Vn) were found to be non-significant and for the majority of these estimates (5/6 comparisons) *D*_est_ = 0, indicating a lack of genetic differentiation between these populations near the center of the zone (Table 2). Permuting the geographic distances between populations determined that the true *rho* values for genetic distance vs. geographic distance lie outside the confidence interval generated from randomly-sampled *rho* values (95% CI: -0.337 - 0.333), affirming the observed relationship between genetic and geographic distance is not a random effect. A positive relationship between pairwise genetic distance vs. geographic distance was also apparent after excluding the non-breeding Sevilla population (rho = 0.377, *P* = 0.040). 

### Hierarchical structuring of genetic variance

No evidence of hierarchical structuring of genetic variation was found by grouping the populations by three migratory directions and three migratory distances (all *F*-statistics *P* > 0.05; Table S4). These results indicate the underlying genetic structure among populations is more complex than simply classifying populations into three migratory orientations and three migratory distances. HierFstat analysis was additionally repeated by categorizing the Norwegian population (Os) as SE migrants (as opposed to SW migrants), but similarly resulted in weak genetic structure; marginally better support for underlying genetic structure was found by categorizing this population as SW migrants (i.e. slightly higher *F*-statistics, albeit still non-significant). 

## Discussion

### Genetic structure among Blackcap populations

Overall, genetic differentiation across all the sampled populations was low and Bayesian clustering, pairwise comparisons and hierarchical analyses all failed to show consistent, significant genetic structure among populations corresponding to different migratory behaviors. A positive relationship was found between genetic distance and (longitudinal) geographic distance of population pairs of Blackcaps located on opposing sides of the SW/SE migratory divide. This result is consistent with the historical isolation of western and eastern breeding populations in Europe previously restricted to potential glacial refugia on the Iberian and Balkan peninsulas, respectively [2,7,54]. Despite this observed relationship between genetic and geographic distance, the only SW/SE pairwise *D*_est_ comparison found to be significantly differentiated was between Ub and Km (over 2000km apart). Such results indicate a lack of neutral genetic divergence between populations of SW and SE migrants and, in fact, many populations within/near the approximated SW/SE contact zone cannot be differentiated with our marker set. We suggest therefore that the SW/SE migratory divide is an ineffective barrier to neutral gene flow across this zone of contact. 

Although isolation-by-distance appears to be maintained among eastern populations, this pattern is not found among western populations and is potentially confounded by the occurrence of sympatrically-breeding populations of NW and SW migrants (as observed in Freiburg, DE). Indeed, NW vs. SW *D*_est_ comparisons were found to significantly differ from SW vs. SE comparisons, confirming beliefs that different dynamics of genetic divergence occur with the NW/SW migratory polymorphism and across the SW/SE migratory divide. In southern Germany (i.e. Freiburg), weak, albeit significant, genetic differentiation was found between NW and SW migrants breeding sympatrically, corroborating results of a previous study by Rolshausen et al. [20], who suggest that only low levels of genetic divergence may be needed to maintain differences in migratory behavior. 

The low nuclear genetic differentiation found across the SW/SE migratory divide in the Blackcap is consistent with similar patterns found across migratory divides in Eurasian Reed Warblers (*Acrocephalus scirpaceus*) in central Europe [31] and Willow Warblers (*Phylloscopus trochilus*) in Scandinavia [12]. A lack of neutral genetic differentiation was similarly found across the Red-billed Quelea (*Quelea quelea*) migratory divide in Africa [10] and between populations of Black-throated Blue Warblers (*Setophaga caerulescens*) with different migratory routes in North America [55]. In addition, complete introgression has recently occurred between populations of Savi’s Warblers (*Locustella luscinioides*) in Iberia despite Pleistocene divergence and long periods of geographic isolation between populations [56]. Without sufficient contemporary reproductive barriers it thus seems that interbreeding often occurs across migratory divides, regardless of historical geographic divergence between populations. 

Potentially, NW migrant Blackcaps may also contribute to breeding populations in other areas in central Europe (e.g. in upper Austria, see 27,57 and Czech/Slovak Republics, see 35). Our pairwise *D*_est_ comparisons suggest the Fr_NW_ population is most genetically similar to the Kf population (i.e. the least genetic distance was found among Fr_NW_ comparisons). This would support the hypothesized origination of NW migrants from within the SW/SE contact zone, first proposed by Helbig [27] from observations of hand-raised Blackcaps from Linz, AU (~25km SW of Kefermarkt) orienting SW-NW directions (mean 268°) during the autumn migratory period. Alternatively, since our *δ* ^2^H assignment method did not indicate a presence of NW migrants in the Kefermarkt population, this result may indicate that the location of the Blackcap migratory divide has shifted eastward; the low genetic divergence found between Fr_NW_ vs. Vn supports this hypothesis, potentially being driven by the expansion of SW migrants into eastern European populations, as proposed previously by Wesołowski [58]. 

Isolation-by-distance (IBD) was found to be non-significant among the 11 breeding populations (after excluding Sevilla). However, patterns of IBD differed on either side of the SW/SE migratory divide: significant IBD was evident among eastern populations (with greater levels pairwise genetic divergence), but not among western populations (with lower levels of pairwise genetic divergence); this result is consistent with the genetic structure of Blackcap mtDNA by Pérez-Tris et al. [28], who found no relationship between genetic and geographic distance among western continental populations (although results may be influenced by the different sets of populations sampled in each study). The relationships between geographic and genetic distance were similarly found to contrast among populations of Eurasian Reed Warblers (*Acrocephalus scirpaceus*) located on opposing sides of their migratory divide in central Europe [31]. Such contradictory patterns geographically spanning migratory divides may be indicative of different ecological and/or evolutionary dynamics acting on opposing sides of migratory divides in central Europe. Unlike that found for eastern Blackcap populations, the lack of IBD demonstrated among western Blackcap populations (i.e. both SW and NW migrants) suggests a mechanism other than geographic isolation may be promoting genetic divergence among western Blackcap populations in the face of gene flow. 

### Mechanisms promoting / preventing genetic divergence

Our results provide little evidence that the historical migratory divide in central Europe provides sufficient reproductive isolation to influence mating preferences based on migratory orientation in the Blackcap. Rather, it appears that interbreeding between SW and SE migrants (and potentially also NW migrants) results in high levels of gene flow across the migratory divide where populations come into contact. Asymmetrical gene flow across the SW/SE migratory divide (i.e. SW migrant Blackcaps outcompeting SE migrants) has been argued to be responsible for recent demographic changes at Polish breeding grounds (e.g. advanced arrival time and increased population size)[58]; interbreeding between Blackcaps with different migratory strategies could therefore potentially occur across an even larger area, resulting not in a divide *per se*, but a gradual transition between migratory orientations across central Europe. Our results indicate an absence of NW migrants in the Białowieża population (from isotope analysis), but cannot exclude the occurrence of SW migrants occurring at this location. In fact, low levels of genetic divergence are found between Białowieża Blackcaps and several SW migrant populations (Ub, Fr_SW_, Mn) as well as SE migrant populations (Kf, Vn, Ry), suggesting Białowieża birds are genetically similar to many other European population due to introgression. Additionally, the distribution of claw *δ* ^2^H from Białowieża birds is roughly consistent with the *δ* ^2^H distribution of Freiburg SW migrants (although we are unable to distinguish SW from SE migrants based solely on claw *δ* ^2^H). Our results, together with those of Wesołowski [58], may provide evidence for either an eastward shift in the geographic position of the divide/contact zone (i.e. asymmetrical hybridization) or a widening of the zone in both east and west directions (i.e. symmetrical hybridization) [59,60]. While further studies are needed to better characterize the dynamics of this zone, movement of the SW/SE contact zone remains plausible for Blackcaps.

Recent studies are now utilizing geolocators to record the annual cycle of long-distance passerine migrants, including migratory routes, durations, and strategies [61,62], as for example in the Swainson’s Thrush (*Catharus ustulatus*) where the existence of a migratory divide in North America has been found [63]. At this narrow migratory divide, low frequencies of Swainson’s Thrush hybrids may be suggestive of post-mating isolation, while evidence for pre-mating isolation (e.g. ecological selection and difference in arrival time) indicates an important role for reinforcement in the maintenance of this hybrid zone which spans a migratory divide [14,19]. Similarly, in Blackcaps both pre- and post-mating isolating mechanisms may likely influence genetic divergence among populations. Crosses between SW and SE migrant Blackcaps produced viable F1 hybrids with intermediate (southerly) orientations [64], but wild-caught Blackcaps from the center of the contact zone (Linz, AT) were found to orient SW-NW (mean 268°) suggesting selection against S-SE orientations (and thus selection against hybrid or SE x SE pairings) at this location [27]. Still, 58% of Blackcaps ringed in the Czech Republic and Slovakia were recovered at SE locations during the non-breeding period [35]. Nevertheless, within the SW/SE contact zone, female preference for males with SE orientations could be responsible for eastward introgression of SW migrant Blackcaps into eastern breeding populations (e.g. Poland) [58].

Rapid genetic divergence between sympatric NW and SW migrant Blackcaps may be promoted by pre-mating isolation. Temporal isolation in spring arrival to breeding grounds in southern Germany has been documented between NW and SW migrants [17,18,30] and presents also an effective isolating mechanism among Swainson’s Thrushes breeding in North America [19]. Such phenological changes can be further affected by global climate change which is impacting the phenology of migratory birds and influencing the advancement of spring migration and arrival to the breeding grounds [58,65,66]. It remains unclear however, if such temporal divergence is locally adaptive in sympatry only, or if NW and SW migrants reflect significant divergence also in allopatry.

We not only find evidence of significant genetic differentiation between NW and SW migrant Blackcaps in sympatry, but NW migrants were significantly differentiated from other populations of SW migrants in western Europe. The observed divergence between NW and SW migrants may be the consequence of a founder effect resulting from a small number of genetically distinct individuals initially establishing the NW migratory route. Allopatric genetic differentiation demonstrated between Fr_NW_ and SW migrants (i.e. Sv and Ub) may indicate that such divergence is not locally adaptive, but that this polymorphism has evolved repeatedly, independent of adaptive pressures on the breeding ground. The behavioral, morphological, and genetic traits observed to be diverging in the Blackcap may be part of a ‘migratory syndrome’ or ‘migratory gene package’ in this species whereby a suite of correlated traits that evolve together are targeted by correlational selection [67–69]. Whether such syndromes are common and generalized among migratory birds may be debatable [70], but provide an exciting frontier to explore. 

Although our Bayesian clustering and hierarchical analyses failed to find significant genetic structure among populations corresponding to the different migratory behaviors investigated, significant (but weak) genetic structure in mtDNA was found among eastern (including SE migrants) and western Blackcap populations (including SW and NW migrants) in a previous study (although only two eastern populations were considered)[28]. Maternally-inherited mtDNA is less likely to introgress across a contact zone than autosomal DNA [71], and reflects only uni-parental gene flow which limits interpretation among populations with highly dynamic migratory behaviors. Migratory orientation may instead be controlled by very few genes [22,24], and the possibility that one or few of microsatellite loci used in our study are linked to functional loci cannot be ruled out, though we did not find any evidence using outlier detection methods (data not shown). Although microsatellites were found to be undifferentiated across the Willow Warbler migratory divide, two AFLPs were shown to exhibit clinal variation corresponding to phenotypic differences [12]. Developing large sets of genetic markers spanning the entire genome and searching for informative markers associated with migratory behavior will provide better resolution in the genetic variation and structure of recently divergent populations, such as the Blackcap [69]. Furthermore, combining molecular and stable isotope markers (e.g. see 72) with data obtained from ring recoveries and geolocators may greatly improve the spatial resolution of species’ movements and thus provide a better understanding of the ecology and evolution of migratory divides. 

## Supporting Information

Figure S1 **Stable isotope assignments.** Claw *δ* ^2^H was used to categorize Spring 2011 Blackcaps caught at four locales (**Ub**, **Fr**, **Kf**, **Bw**) as inhabiting northern wintering grounds on the British Isles (i.e. NW migrants) or southern wintering grounds on Iberian or Balkan Peninsulas (i.e. SW or SE migrants) based on reference claw *δ* ^2^H from British Isles and Spain, respectively (see Methods). (A) Isotope assignment results per population. Uebersyren, LU (Ub): NW = 1, SW = 42; Freiburg, DE (Fr): NW = 23, SW = 176, Unassigned (?) = 10; Kefermarkt, AT (Kf): South = 105; Białowieża, PL (Bw): South = 61. (B) Distribution of raw claw *δ* ^2^H isotopes from each population, Ub = 61; Fr = 209; Kf = 105; Bw = 61. Claw *δ* ^2^H from **Ub**, **Kf**, and **Bw** was found to significantly differ from **Fr** *δ* ^2^H distribution (Wilcoxon Rank Sum Test *P* < 0.05), while only **Kf** and **Bw** were found to significantly differ among remaining comparisons. (C) *δ* ^2^H Distribution of *N* = 23 NW migrants and *N* = 82 SW migrants selected from Freiburg, Fr population for genetic analyses Width of bars are proportional to sample size.
(TIF)

Table S1 **Characteristics of 14 microsatellite markers.** Accession No.: GenBank accession numbers; *N* alleles: number of alleles per marker; Ho / He: Observed / Expected Heterozygosities; HWE by pop: populations that were found to deviate significantly for HWE; Null Allele Freq: mean frequency of null alleles per marker.
(DOCX)

Table S2 **Allele frequency comparison over populations.**
(XLS)

Table S3 **Summary of STRUCTURE results.** **#K**: number of clusters; **Mean LnP(*K*)**: mean posterior probability of given *K*; **Stdev LnP(*K*)**: standard deviation of mean posterior probability of given *K*.
(DOCX)

Table S4 **F-statistics of hierarchical structuring of genetic variance.** Relationship between hierarchy levels are indicated in rows with ‘/’ indicating ‘within’; **Hierarchy A**: 3 migratory directions (NW, SW, SE) and 3 migratory distances (short-, intermediate-, long-distance) with the **Os** population characterized having SW migratory direction (dir); **Hierarchy B**: 3 migratory directions (NW, SW, SE) and 3 migratory distances (short-, intermediate-, long-distance) with the **Os** population characterized having SE migratory direction (dir).
(DOCX)
