# Supplementary figures and images for: Contrasting Patterns of Genetic Differentiation among Blackcaps (Sylvia atricapilla) with Divergent Migratory Orientations in Europe
Source: PLoS One. 2013 Nov 21;8(11):e81365. doi: 10.1371/journal.pone.0081365 (PMC3836794; doi:10.1371/journal.pone.0081365)

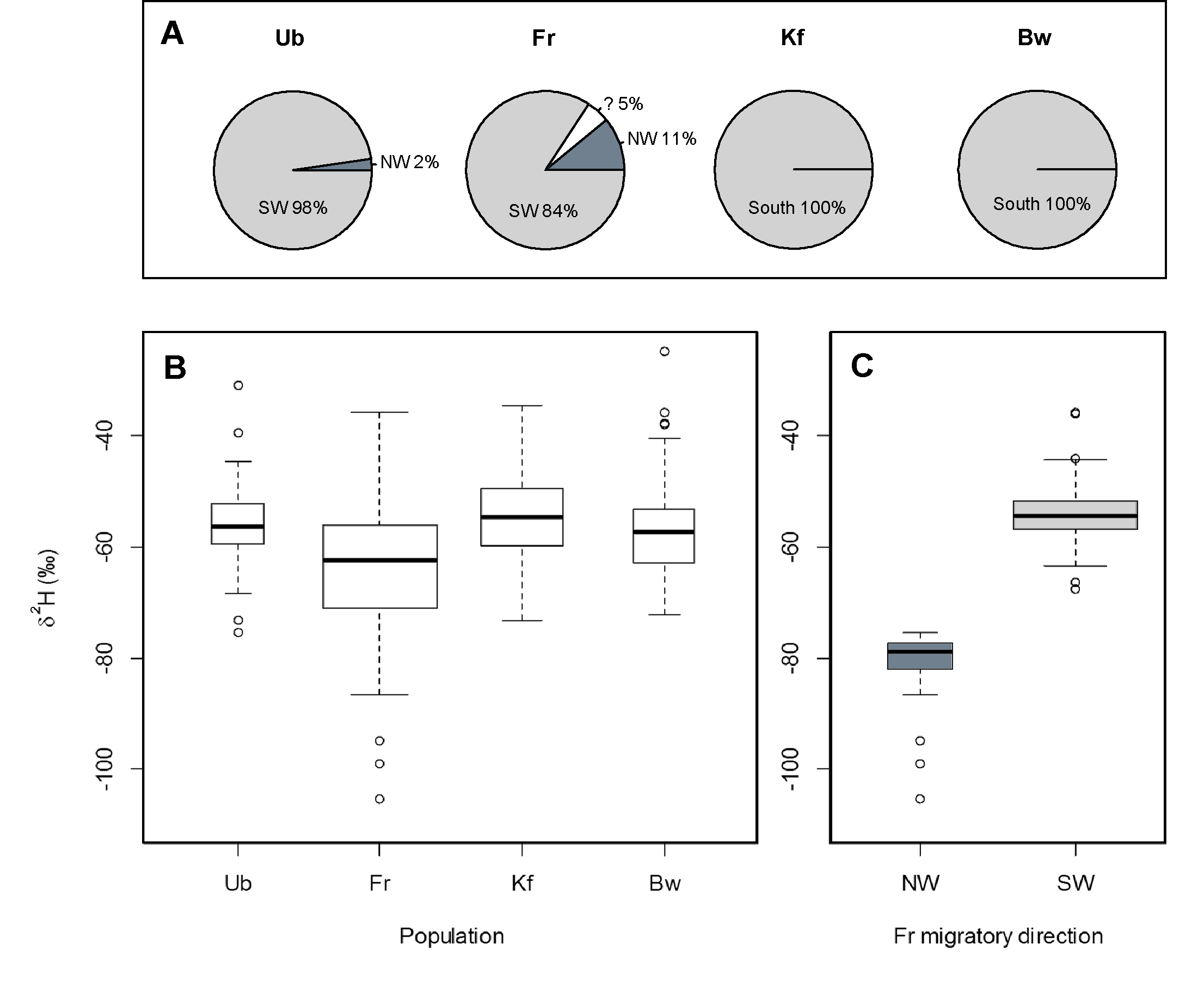

Supplement: Figure S1 — Stable isotope assignments. Claw δ 2H was used to categorize Spring 2011 Blackcaps caught at four locales (Ub, Fr, Kf, Bw) as inhabiting northern wintering grounds on the British Isles (i.e. NW migrants) or southern wintering grounds on Iberian or Balkan Peninsulas (i.e. SW or SE migrants) based on reference claw δ 2H from British Isles and Spain, respectively (see Methods). (A) Isotope assignment results per population. Uebersyren, LU (Ub): NW = 1, SW = 42; Freiburg, DE (Fr): NW = 23, SW = 176, Unassigned (?) = 10; Kefermarkt, AT (Kf): South = 105; Białowieża, PL (Bw): South = 61. (B) Distribution of raw claw δ 2H isotopes from each population, Ub = 61; Fr = 209; Kf = 105; Bw = 61. Claw δ 2H from Ub, Kf, and Bw was found to significantly differ from Fr δ 2H distribution (Wilcoxon Rank Sum Test P < 0.05), while only Kf and Bw were found to significantly differ among remaining comparisons. (C) δ 2H Distribution of N = 23 NW migrants and N = 82 SW migrants selected from Freiburg, Fr population for genetic analyses Width of bars are proportional to sample size. (TIF) [file pone.0081365.s001.tif]
