# Supplementary material for: Contrasting Patterns of Genetic Differentiation among Blackcaps (Sylvia atricapilla) with Divergent Migratory Orientations in Europe
Source: PLoS One. 2013 Nov 21;8(11):e81365. doi: 10.1371/journal.pone.0081365 (PMC3836794; doi:10.1371/journal.pone.0081365)
Supplement: Table S1 — Characteristics of 14 microsatellite markers. Accession No.: GenBank accession numbers; N alleles: number of alleles per marker; Ho / He: Observed / Expected Heterozygosities; HWE by pop: populations that were found to deviate significantly for HWE; Null Allele Freq: mean frequency of null alleles per marker. (DOCX) [file pone.0081365.s002.docx]

**Table S1.** Characteristics of 14 microsatellite markers. **Accession No**.: GenBank accession numbers. ***N* alleles**: number of alleles per marker. **Ho / He**: Observed / Expected Heterozygosities. **HWE by pop**: populations that were found to deviate significantly for HWE. **Null Allele Freq**: mean frequency of null alleles per marker.
