# Supplementary material for: Contrasting Patterns of Genetic Differentiation among Blackcaps (Sylvia atricapilla) with Divergent Migratory Orientations in Europe
Source: PLoS One. 2013 Nov 21;8(11):e81365. doi: 10.1371/journal.pone.0081365 (PMC3836794; doi:10.1371/journal.pone.0081365)
Supplement: Table S3 — Summary of STRUCTURE results. #K: number of clusters; Mean LnP(K): mean posterior probability of given K; Stdev LnP(K): standard deviation of mean posterior probability of given K. (DOCX) [file pone.0081365.s004.docx]

**Table S3.** Summary of STRUCTURE results

**#*K***: number of clusters; **Mean LnP(*K*)**: mean posterior probability of given *K*; **Stdev LnP(*K*)***:* standard deviation of mean posterior probability of given *K*.
