# Supplementary material for: Contrasting Patterns of Genetic Differentiation among Blackcaps (Sylvia atricapilla) with Divergent Migratory Orientations in Europe
Source: PLoS One. 2013 Nov 21;8(11):e81365. doi: 10.1371/journal.pone.0081365 (PMC3836794; doi:10.1371/journal.pone.0081365)
Supplement: Table S4 — F-statistics of hierarchical structuring of genetic variance. Relationship between hierarchy levels are indicated in rows with ‘/’ indicating ‘within’; Hierarchy A: 3 migratory directions (NW, SW, SE) and 3 migratory distances (short-, intermediate-, long-distance) with the Os population characterized having SW migratory direction (dir); Hierarchy B: 3 migratory directions (NW, SW, SE) and 3 migratory distances (short-, intermediate-, long-distance) with the Os population characterized having SE migratory direction (dir). (DOCX) [file pone.0081365.s005.docx]

**Table S4.** F-statistics of hierarchical structuring of genetic variance.

Relationship between hierarchy levels are indicated in rows with ‘/’ indicating ‘within’; Hierarchy A: 3 migratory directions (NW, SW, SE) and 3 migratory distances (short-, intermediate-, long-distance) with the Os population characterized haveing SW migratory direction (dir); Hierarchy B: 3 migratory directions (NW, SW, SE) and 3 migratory distances (short-, intermediate-, long-distance) with the Os population characterized haveing SE migratory direction (dir).
